# Associations Between Cognitive Functions and Physical Frailty in Patients With Parkinson’s Disease

**DOI:** 10.3389/fnagi.2019.00283

**Published:** 2019-10-30

**Authors:** Wei-Che Lin, Yu-Chi Huang, Chau-Peng Leong, Meng-Hsiang Chen, Hsiu-Ling Chen, Nai-Wen Tsai, Hui-Hsin Tso, Po-Cheng Chen, Cheng-Hsien Lu

**Affiliations:** ^1^Department of Diagnostic Radiology, Kaohsiung Chang Gung Memorial Hospital, Chang Gung University College of Medicine, Kaohsiung, Taiwan; ^2^Department of Physical Medicine and Rehabilitation, Kaohsiung Chang Gung Memorial Hospital, Chang Gung University College of Medicine, Kaohsiung, Taiwan; ^3^Department of Neurology, Kaohsiung Chang Gung Memorial Hospital, Chang Gung University College of Medicine, Kaohsiung, Taiwan

**Keywords:** cognitive impairment, executive function, physical frailty, Parkinson’s disease, movement disorder

## Abstract

**Background**: Parkinson’s disease (PD) is a neurodegenerative disease manifested by both motor and non-motor dysfunctions and co-existence of cognitive impairment and physical frailty is common. Given that research in this area is limited, a better understanding of associated factors with physical frailty could provide a focused screening method and facilitate early intervention in PD.

**Methods**: Seventy-six patients with idiopathic PD were recruited and Fried’s criteria of physical frailty were used to group all participants. Comprehensive cognitive tests and clinical characteristics were measured, and univariate and multivariate analysis was performed to explore the relationship between clinical factors or neuropsychological functions.

**Results**: Twenty-nine patients with PD (38%) exhibited physical frailty. Compared to PD patients without frailty, PD patients with frailty were older in age and demonstrated worse disease severity and poorer cognitive functions, including attention, executive function, memory, speech and language, and visuospatial function (*p* < 0.05). Further, stepwise logistic regression analysis revealed that disease severity by the Unified Parkinson’s Disease Rating Scale (UPDRS) total score (OR: 1.065; 95% CI: 1.033–1.099) and executive function (OR: 0.724; 95% CI: 0.581–0.877) were independent risk factors for predicting physical frailty (*p* = 0.003 and 0.002). The best cut-off points are 46 in UPDRS (sensitivity: 62.1%; specificity: 91.5%).

**Conclusions**: Executive function impairment is an independent risk factor for the development of physical frailty with disease progression. Awareness of such comorbidity might provide a screening tool to facilitate investigation in their underlying etiology and early intervention for frailty prevention.

## Introduction

Parkinson’s disease (PD) is one of the most common neurodegenerative diseases (de Lau and Breteler, 2006). Incidence rates of PD were reported as 8–18 per 100,000 person-year and its prevalence is estimated at 0.3% in the general population and 1% in older people aged greater than 60 years (de Lau and Breteler, 2006). The cardinal motor features in PD, including tremor, bradykinesia, rigidity, and postural instability frequently lead to abnormal limbs or trunk postures, impaired gait ability, and decreased physical activity (Jankovic, 2008). Physical frailty is also a common presentation in patients with PD and is significantly associated with morbidity and mortality (Ahmed et al., 2008). However, limited evidence or studies are available on the relationships between physical frailty and clinical characteristics or functional capabilities in PD. In addition to physical frailty, non-motor symptoms, such as cognitive impairment and dementia, psychosis, mood disorders, and fatigue (Jankovic, 2008; Barone et al., 2009), are common comorbidities as the disease progresses. It is reasonable to suggest that the possible interaction between the decline in some of the motor and non-motor functions in PD may be important factors in physical frailty. Clinically, it is important to identify the predicting factors for physical frailty in early stage PD and provide appropriate management to prevent further disease progression.

Frailty is a common clinical syndrome in older adults due to aging-related decline in multiple physiological systems. According to the reports of SHARE and BLSA-II, the prevalence of physical frailty among community-dwelling elderly is 17% in Europe and 12.3% in China (Santos-Eggimann et al., 2009; Zheng et al., 2016). Indeed, frailty is associated with negative health outcomes, including mobility disability, deterioration of daily living activities, institutionalization, and mortality (Xue, 2011). In proposing the term frailty, Fried et al. (2001) operationalized five phenotypes of low energy, decreased grip strength, slow walking speed, unintentional weight loss, and low physical activity. Subsequently, the Frailty Index of Accumulative Deficits (FI-CD), which was proposed by Rockwood and Mitnitski, took into account the multidimensional nature of frailty and included psychosocial domains such as cognitive decline and other psychosocial factors, as well as geriatric syndromes, in its definition of frailty (Rockwood et al., 2005). However, the mathematical nature of the FI-CD, as well as its being more time consuming to complete, have led to it being relatively unpopular in clinical application (Dent et al., 2016). In a review article that considered eight studies that focused on PD and frailty, most of the included studies used Fried’s criteria, while only one study used the FI-CD (Smith et al., 2019). Additional research has demonstrated that elderly with physical frailty exhibited poorer cognitive performance and greater cognitive deterioration than individuals without frailty (Boyle et al., 2010; Rogers et al., 2017). On the other hand, individuals with cognitive dysfunction or dementia showed a higher risk of physical frailty (Robertson et al., 2013). Thus, specific domains of cognitive function among the elderly might even associate with higher risk of frailty onset and act as a predictor of mortality and disability (Gross et al., 2016; Rosado-Artalejo et al., 2017).

PD is recognized as a complex condition with neuropsychiatric and non-motor symptoms in addition to its motor symptomatology (Langston, 2006). However, the neuropathological and neuropsychological manifestations were not well understood and heterogeneous among PD patients with cognitive impairment. The general pattern of cognitive impairment in PD was illustrated by memory and executive dysfunctions (Muslimovic et al., 2005) and often presented in the early stages of PD. Besides, PD patients with cognitive impairments had worse motor symptoms, including postural instability and gait disorder (Sollinger et al., 2010). In addition, PD patients with mild cognitive impairment (PD-MCI) had a higher risk of developing PD dementia (PDD) with declined premorbid level that interfered with daily activities and were associated with reduced quality of life, increased risk of institutionalization, and mortality (Hobson and Meara, 2004; Perez et al., 2012). For instance, Ahmed et al. (2008) found that frail PD patients demonstrated higher Unified Parkinson’s Disease Rating Scale (UPDRS) scores, which covered mentation, behavior, mood, daily living ability, and motor capacity. Since aging increases the risk of PD and the risk of developing dementia in PD is approximately five to six times greater than the general population (Hobson and Meara, 2004), we considered that a higher prevalence of cognitive dysfunction, particularly in some specific domain deficits may be associated with a higher incidence of physical frailty in PD patients throughout the disease progression.

In this study, we aim to determine the independent predictors of physical frailty from age, disease severity/duration, and global or specific domains of cognitive dysfunction in patients with PD. A better understanding of associated factors with physical frailty could provide a focused screening method and facilitate early intervention in patients with PD.

## Materials and Methods

### Study Design

The aim of this case-control study was to identify the relationship between cognitive function and physical frailty in patients with PD. The study was approved by the institutional review board of the hospital. All investigators performed the procedures according to the ethical principles for medical research involving human subjects.

### Participants

Seventy-six patients (35 males and 41 females; mean age: 62.64 ± 9.23 years) with idiopathic PD diagnosed according to the United Kingdom Brain Bank criteria (Ramaker et al., 2002) and without other neurological or psychiatric disease were prospectively enrolled at the Neurology Department of Chang Gung Memorial Hospital, a tertiary medical center in Taiwan. Those patients with idiopathic PD were treated and regularly follow up in the hospital. The inclusion criteria were patients with idiopathic PD and aged between 40 and 75 years. Patients with the following conditions were excluded: atherosclerotic narrowing on intracranial and extracranial vessels (>50% stenosis) with or without evidence of old cerebral infarctions, coronary artery diseases status post-percutaneous transluminal coronary angioplasty or bypass surgery, renal failure requiring hemodialysis or peritoneal dialysis, moderate to severe heart failure (New York Heart Association class III and IV), and central or peripheral disorders known to affect autonomic nervous systems. For each PD patient, all assessments were conducted in the ON-state. Informed written consent was obtained from all patients prior to the start of the study procedures.

### Methods

All PD patients were screened for physical frailty using Fried’s criteria (Fried et al., 2001) which are practical measurement criteria that have been applied in multiple epidemiological studies (Fried et al., 2001; Bandeen-Roche et al., 2006; Gill et al., 2010; Lee et al., 2015). Five components, including unintentional weight loss, exhaustion, low grip strength, slowness while walking, and low levels of activity, were measured in all enrolled patients. According to this criteria of frailty, following are the evaluation steps (Fried et al., 2001; Bieniek et al., 2016): first, the information about unintentional weight loss in the last year was reported; then, declined level of exhaustion and lowered physical activity were ranked by the caregivers; third, a dynamometer was used to measure the grip strength, and lastly, gait speed was measured while walking over a 10-m walkway. Participants who met 0–2 of these criteria were allocated into non-frail group, and those who met at least three of these five criteria were categorized into frail group.

### Clinical Demography and Severity of PD

Clinical characteristics for these patients with idiopathic PD including age, gender, duration since disease onset or diagnosis, and medicine duration, were evaluated. Each PD patient’s disease severity and functional status were evaluated by the UPDRS (Ramaker et al., 2002). The sections of UPDRS consist of the following: (1) an evaluation of mentation, behavior, and mood; (2) an evaluation of daily activities regarding speech, salivation, swallowing, handwriting, cutting food and handling utensils, dressing, hygiene, turning in bed, falling, freezing when walking, walking, tremor, and sensory complaints; and (3) motor capability. Higher scores represent more severe symptoms of PD. The modified Hoehn & Yahr Staging Scale (Ramaker et al., 2002) were used to evaluate the severity of PD based on clinical presentations and functional ability from stages 1–5 (higher levels indicating higher severity of the disease). The Schwab and England Activities of Daily Living (ADL) scale (Hobson and Meara, 2004) was used to assess a person’s daily function for PD, in which a score of 100% indicates complete independence and 0% indicates a bedridden status with vegetative functions.

### Neuropsychological Assessments

A neuropsychological battery of tests was performed by one clinical psychologist blinded to each patient’s status. These tests covered five domains: attention, executive function, speech and language, memory, and visuospatial function (Chen et al., 2017). Attention was assessed using the digit span score in the Wechsler Adult Intelligence Scale-III (WAIS-III; Taylor and Heaton, 2001) and the attention and orientation scores from the Cognitive Ability Screening Instrument (CASI; Teng et al., 1994). Executive function was measured using the similarities, arithmetic, matrix reasoning, picture arrangement, and digit symbol coding scores in the WAIS-III and the abstract thinking and judgment score in the CASI. Speech and language function was evaluated using the vocabulary and comprehension scores in the WAIS-III and the language score in the CASI. Memory function was assessed using the information score in the WAIS-III and the short- and long-term memory scores in the CASI. Visuospatial function was assessed using the picture completion and block design scores from the WAIS-III and the drawing score from the CASI.

### Statistical Analysis

The demographic data were compared among the study groups using the two-sample Mann–Whitney *U* test and the Pearson chi-square test, where appropriate. The predictive relation among different clinical characteristics and physical frailty was analyzed using stepwise logistic regression. Due to the different scoring systems and numbers of sub-tests in each domain of the neuropsychological testing, the scores of each subtest in neuropsychological assessments were converted into a *z*-score, and then summed up into five main cognition categories. Following, univariate logistic regression was used to analyze the correlation between each clinical characteristic and physical frailty. Any variable with a *p*-value of <0.1 in univariate regression was selected to be analyzed using multivariate logistic regression. Further, a forward conditional method without multicollinearity was chosen while performing multivariate logistic regression. Receiver operating characteristic curve (ROC curve) was used to find the best cut-off point of risk factors and further analyze the sensitivity and specificity of the best cut-off point. Statistical significance was defined as a *p*-value of <0.05. All statistical tests were performed using SPSS 19.0 (SPSS, Inc., Chicago, IL, USA).

## Results

### Clinical Characteristics in PD With/Without Frailty

Table 1 presents the clinical characteristics in idiopathic PD patients with and without frailty. There were 29 PD patients (38.2%) with physical frailty (12 men and 17 women; mean age: 65.34 years) and 47 PD patients (61.8%) without physical frailty (23 men and 24 women; mean age: 60.98 years). A significant difference was found in age between PD patients with and without frailty (65.34 ± 8.42 years and 60.98 ± 9.45 years, *p* = 0.033). However, while longer disease duration (2.74 years vs. 2.12 years) and medication duration (1.64 years vs. 1.07 years) were found in the PD patients with frailty compared to those without, the differences were not significant. As for the equivalent doses of levodopa, a statistically significant between-group difference was observed (326.83 ± 276.29 mg/day and 547.77 ± 289.37 mg/day, *p* = 0.001). The mean total scores of UPDRS were significantly higher in PD patients with frailty compared with PD patients without frailty (48.96 vs. 26.17, *p* = 0.001). The mean stages in modified Hoehn & Yahr staging in PD patients with and without frailty were 2.17 and 1.54, respectively (*p* = 0.009), and the Schwab and England ADL scales in PD patients with and without frailty were 79.31 and 86.8, respectively (*p* = 0.006).

**Table 1:** Clinical characteristics in Parkinson’s disease patients without and with frailty.

	PD without frailty (*n* = 47)	PD with frailty (*n* = 29)	*P*
Gender (male, female)	23, 24	12, 17	0.637
Age [years, mean (SD)]	60.98 (9.45)	65.34 (8.42)	0.033*
Disease duration [years, mean (SD)]	2.12 (2.70)	2.74 (2.82)	0.136
Medicine duration [year, mean (SD)]	1.07 (1.77)	1.64 (2.09)	0.157
Equivalent doses of levodopa [mg/day, mean (SD)]	326.83 (276.29)	547.77 (289.37)	0.001**
UPDRS I, mean (SD)	2.91 (2.10)	4.06 (2.72)	0.076
UPDRS II, mean (SD)	7.02 (5.08)	12.34 (6.98)	0.001**
UPDRS III, mean (SD)	18.13 (12.52)	32.55 (14.52)	0.001**
UPDRS total, mean (SD)	26.17 (14.95)	48.96 (22.08)	0.001**
Modified Hoehn & Yahr scale, mean (SD)	1.54 (1.02)	2.17 (1.12)	0.009**
Schwab and England ADL scale, mean (SD)	86.80 (16.70)	79.31 (15.10)	0.006**

In clinical characteristics, gender was analyzed by using chi-square. Age, disease duration, duration of taking medicines, equivalent doses of levodopa, UPDRS, Hoehn & Yahr scale, Schwab and England ADL scale, and MMSE were analyzed by using the Mann–Whitney *U* test. UPDRS, the Unified Parkinson’s Disease Rating Scale; MMSE, Mini-Mental State Examination; *P < 0.05, **P < 0.01.

### Group Comparisons of Neuropsychological Assessments

In the neuropsychological assessments, the PD patients with frailty had significantly poor performances in attention (digit span and attention: *p* = 0.001–0.005), executive function (digit symbol coding, similarities, arithmetic, letter number sequencing, matrix reasoning, and abstract thinking: *p* = 0.001–0.002), memory (short-term memory and information: *p* = 0.004 and *p* = 0.001), speech and language (vocabulary, comprehension, and language: *p* = 0.001–0.003), and visuospatial function (picture completion, block design, and drawing: *p* = 0.003–0.03). However, this was not the case in the orientation of attention and in long-term memory (Table 2).

**Table 2:** The comparisons of the neuropsychological assessments between Parkinson’s disease patients without and with frailty.

	PD without frailty (*n* = 47)	PD with frailty (*n* = 29)	*P*
**Attention Function**
Digit span, mean (SD)	10.19 (2.68)	8.28 (2.90)	0.005**
Attention, mean (SD)	7.36 (0.82)	6.38 (1.21)	0.001**
Orientation, mean (SD)	16.85 (2.44)	16.41 (2.44)	0.239
**Executive Function**
Digit symbol coding, mean (SD)	8.78 (2.84)	5.34 (2.55)	0.001**
Similarity, mean (SD)	9.68 (2.61)	7.59 (2.54)	0.001**
Arithmetic, mean (SD)	9.30 (2.99)	6.76 (1.64)	0.001**
Letter number sequencing, mean (SD)	9.44 (2.91)	6.74 (3.29)	0.002**
Matrix reasoning, mean (SD)	9.47 (3.06)	6.41 (2.43)	0.001**
Abstract thinking, mean (SD)	9.43 (1.86)	7.38 (1.94)	0.001**
**Memory Function**
Short-term memory, mean (SD)	9.40 (2.45)	7.18 (3.09)	0.004**
Long-term memory, mean (SD)	9.79 (0.75)	9.52 (1.15)	0.245
Information, mean (SD)	9.51 (2.60)	7.69 (1.89)	0.001**
Speech and Language
Vocabulary, mean (SD)	9.62 (3.46)	7.38 (2.34)	0.003**
Comprehension, mean (SD)	9.78 (3.16)	6.55 (2.38)	0.001**
Language, mean (SD)	9.70 (0.60)	8.73 (1.28)	0.001**
**Visuospatial Function**
Picture completion, mean (SD)	8.94 (3.05)	7.24 (2.56)	0.011*
Block design, mean (SD)	8.47 (3.22)	6.31 (2.17)	0.003**
Drawing, mean (SD)	9.38 (1.48)	8.69 (1.82)	0.030*

Mann–Whitney test was used to analyze the differences of various neuropsychological assessments between two groups, *P < 0.05, **P < 0.01.

### Risk Factors Associated With Physical Frailty

In a univariate analysis for PD patients (Table 3), age, UDPRS scores, Hoehn & Yahr scale, and all five specific domains of the neuropsychological assessments were all significantly related to physical frailty (*p* < 0.05). In a multivariate analysis, variables with multicollinearity (Variance Inflation Factor >10) were excluded for further analysis. Finally, variables, including age, UDPRS scores, Hoehn & Yahr scale, attention function and executive function, were used in the stepwise logistic regression analysis. Only UPDRS total score (OR: 1.065; 95% CI: 1.033–1.099) and executive function (OR: 0.724; 95% CI: 0.581–0.877) were statistically significant for predicting the PD patients with frailty (*p* = 0.003 and 0.002, respectively).

**Table 3:** Univariate and multivariate logistic regression analysis for Parkinson’s disease with frailty.

	Univariate		Multivariate	
	OR (95% C.I.)	P1	OR (95% C.I.)	P2
**Clinical characteristics**				
Age (years)	1.057 (1.000–1.117)	0.048*	-
Sex	0.802 (0.315–2.043)	0.644	-
Disease duration (years)	1.086 (0.918–1.284	0.336	-
Duration of taking medicines (years)	1.013 (0.993–1.034)	0.210	-
UPDRS total	1.065 (1.033–1.099)	0.001**	1.063 (1.019–1.110)	0.005**
Hoehn & Yahr scale	1.690 (1.072–2.663)	0.024*	-	-
Schwab and England ADL scale	0.972 (0.943–1.002)	0.064	-	-
**Neuropsychological assessments**
Attention	0.699 (0.547–0.894)	0.004**	-	-
Executive Function	0.701 (0.581–0.846)	0.001**	0.675 (0.532–0.857)	0.001**
Memory	0.660 (0.500–0.872)	0.003**	-	-
Speech and Language	0.600 (0.459–0.785)	0.001**	-	-
Visuospatial Function	0.717 (0.564–0.912)	0.007**	-	-

The scores of neuropsychological assessments were changed into a z-score, and then summed up into five main cognition categories, which are attention, executive function, memory, speech and language, and visuospatial function (see Table 2 for more details). Univariate logistic regression was used to analyze the correlation between each variable and patients with or without frailty. Any variables that had *p* < 0.1 were selected to be further analyzed by using multivariate logistic regression. In a multivariate analysis, variables with multicollinearity (Variance Inflation Factor >10) were excluded for further analysis. Finally, variables, including age, UDPRS scores, Hoehn & Yahr scale, attention function and executive function (boldface), were used in the stepwise logistic regression analysis. When executing multivariate logistic regression, “forward: conditional” was chosen as our method. MMSE, Mini-Mental State Examination; UPDRS, Unified Parkinson’s Disease Rating Scale, **P < 0.01.

**Figure 1:**
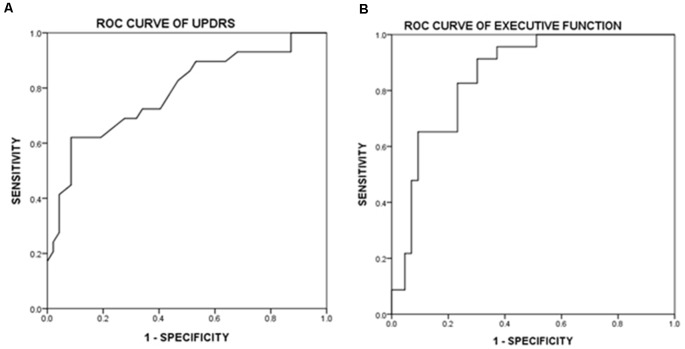
Receiver operating characteristic curve (ROCcurve) of Unified Parkinson’s Disease Rating Scale (UPDRS) and executive function. **(A)** ROC curve of UPDRS. Area under this curve is 0.785 (0.678–0.895). After further calculation, Youden’s index is 0.56 and the best cut-off point is 46, which means if a patient scored higher, then there is a high possibility of having physical frailty. **(B)** ROC curve of executive function. Area under this curve is 0.854 (0.764–0.945). After further calculation, Youden’s index is 0.611 and the best cut-off point is 0.222, which means if *z*-score is lower, then it is highly possible that the patient experience physical frailty.

**Figure 2:**
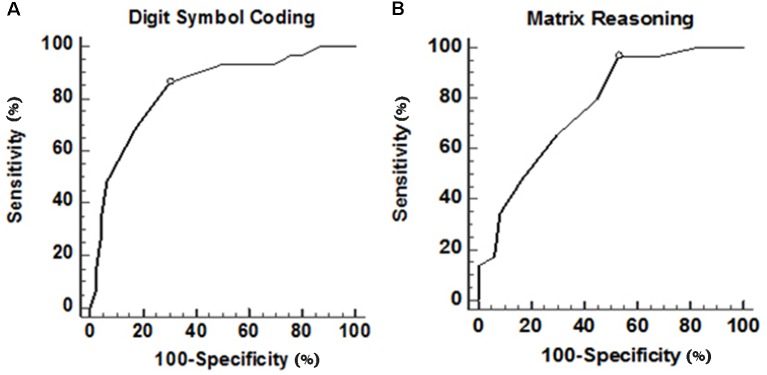
ROC curve of digit symbol coding and matrix reasoning in executive function. **(A)** ROC curve of digit symbol coding. Area under this curve is 0.835 (0.732–0.911). After further calculation, Youden’s index is 0.56 and the best cut-off point is 7.40, which means if a patient scored lower, then there is a high possibility of having physical frailty. **(B)** ROC curve of matrix reasoning. Area under this curve is 0.766 (0.655–0.856). After further calculation, Youden’s index is 0.43 and the best cut-off point is 8.38, which means if scored lower than that, then it is highly possible that the patient experience physical frailty.

In the ROC curve analysis, Figure 1 illustrates that the area under curve (AUC) of UPDRS is 0.785 (acceptable discrimination: 0.7–0.79); AUC of executive function is 0.854 (excellent discrimination: 0.8–0.89). The cut-off points of UPDRS and executive function were 46 and 0.222, respectively. The sensitivity and the specificity at the best cut-off point of UPDRS were 62.1% and 91.5%, respectively. At the best cut-off point of executive function, the sensitivity was 91.3% and the specificity was 69.7%. Further analysis indicated that among those subtests belong to executive function, both digit symbol coding (*p* = 0.021) and matrix reasoning (*p* = 0.036) had more power in predicting frailty, comparatively. The ROC curve showed that AUC of digit symbol coding was 0.835 (excellent discrimination), the AUC of matrix reasoning was 0.766 (acceptable discrimination). The cut-off points of digit symbol coding and matrix reasoning was 7.4 (sensitivity 85.6%, specificity 70.0%) and 8.3 (sensitivity 96.6%, specificity 46.1%; Figure 2).

## Discussion

In the present study, we investigated the associations among age, disease duration, disease severity, cognitive dysfunctions, and physical frailty in PD. Mostly neuropsychological functions, including attention, executive function, memory, speech and language, and visuospatial function, were significantly worse in PD patients with physical frailty than those without. Multivariate analysis revealed that disease severity and executive function were independent risk factors for predicting physical frailty in PD. We found that one *z*-score increment in UPDRS total score increased the risk of physical frailty in PD patients by 6.4% and a decrease in executive function by one *z*-score increased the risk of physical frailty by 38.1%. Our findings partially support previous work demonstrating that PD patients develop frailty over the course of their disease progression (Ahmed et al., 2008) and also highlight the importance of interaction in cognitive-physical integrity (Gross et al., 2016; Rosado-Artalejo et al., 2017).

Frailty is common in older populations and carries a higher risk of poor health status in terms of falls, disability, institutionalization, and mortality (Xue, 2011). Several past studies have reported prevalence rates of frailty in PD patients ranging from 29% to 33% according to the Fried criteria, whereas the prevalence of frailty was found to be higher in research using the FI-CD (50% to 67%; Ahmed et al., 2008; Roland et al., 2012a,b, c, 2014; Buchman et al., 2013; Smith et al., 2019). In another study regarding screening, Ahmed et al. (2008) also found a higher prevalence of physical frailty in PD patients than in the general population, as well as higher UPDRS scores in frail patients. Tan et al. (2018) also reported higher incidences of sacropenia and frailty among PD patients. Otherwise, older age and greater PD motor severity could predict frailty in PD. Indeed, past studies have found that inactivity can induce loss of muscle mass in older adults, and that loss of muscle mass strongly predicted functional impairments and disabilities (Janssen et al., 2002), which may further exacerbate PD-associated neuropathology in the muscles, thus leading to increased weakness and frailty. Postural instability and gait difficulty are the main motor manifestations of PD, and these have been reported to make some contributions to overall disability, especially during the later course of the disease (Jankovic, 2008; Muslimovic et al., 2008). In the past, studies have focused on different factors that might cause various disabilities in PD, and it was considered that older age at onset; higher scores in terms of postural instability, gait disorder, and disease severity; and disease duration may be associated with different disabilities, such as greater postural abnormalities (Post et al., 2007; Muslimovic et al., 2008). In addition, such disturbances in posture, balance, and gait in PD were found to contribute to adverse cognitive outcomes (Schneider et al., 2015). Similarly, in our study, it was also found that there was a higher prevalence of physical frailty (38%) among PD patients of older ages with more severe conditions and receiving larger doses of levodopa. Furthermore, we used ROC analysis to determine that the best cut-off point of the UPDRS is a score of 46, which was found to have a sensitivity of 62.1% and a specificity of 91.5%. Notably, this information has the potential to aid in the development of a screening tool that could be used to identify PD patients at high risk of physical frailty.

Higher incidences of cognitive impairments were also found in PD patients with frailty in the present study. When there is a loss of dopamine in the basal ganglia, negative impacts on not only motor functional impairment but also non-motor symptoms, such as automatic behavior and cognitive function are evident (Petzinger et al., 2013). MCI is common in the early stage of PD, which will impede motor-skill learning and will progress with disease severity (Petzinger et al., 2013). Additionally, MCI has an increased risk of developing into dementia, which often eclipses motor dysfunction as the main reason of becoming disabled, and the occurrence of dementia is considered to be an independent predictor of mortality in PD (Forsaa et al., 2010). Previous functional magnetic resonance imaging (fMRI) studies have demonstrated that in PD patients, both cognitive function and motor function are related to their cortical atrophy and those with cognitive impairment and worse disease severity may experience extensive cortical atrophy and decreased cortical perfusion (Chen et al., 2017). Until now, it is still a chicken-and-egg conundrum for motor and cognitive functions in PD and the analysis of comorbidity with cognitive deficit in PD patients with frailty is still limited. However, we emphasized the importance of recognizing cognitive decline in the early stage of PD, which might be a useful clinical sign for the initiation of frailty prevention with PD patients. We also found that using a *z*-score lower than 0.222 in executive function (sensitivity: 91.3% and specificity: 69.7%) could help physicians to reveal executive impairment in patients with PD to identify those at higher risk of physical frailty.

Another important, but not well explored, finding in the present study is the prediction of frailty by executive function in PD patients. Executive function covers many high-level capabilities required to perform complex activities or tasks. The capabilities are coordinated by the prefrontal area, including its projected cortex and subcortical areas, with a wide network (Rosado-Artalejo et al., 2017). Relevantly, the development of executive function impairments in PD was reasonable since the alternations in both the corticostriatal and mesolimbic pathways are common in PD (Chen et al., 2017). The onset of primary executive dysfunction is related to the risks for general cognitive declines in other domains and affects the performances driven by other cognition functions. Furthermore, executive dysfunction was reported as a specific domain of cognition function that mostly contributed to physical frailty in elderly (Gross et al., 2016). Similarly, we also found that executive function was an important determinant of physical frailty in PD after eliminating the overlapping influences of other cognition functions. Due to the degenerative process of dopaminergic nigrostriatal pathway, motor impairments are progressively declined and cognition dysfunction might be partially depended on the loss of dopaminergic neurons and also associated with a disconnected prefrontal cortex, hippocampus, and amygdala (Solari et al., 2013). The frontal and temporal cortices are in charge of gait control, which shares a similar neuro-mechanism with executive function (Rosado-Artalejo et al., 2017). Likewise, atrophy of both gray and white matter in the prefrontal lobe, temporal areas, and basal ganglions lead to slow gait, muscle mass loss, and weakness (Gross et al., 2016). Therefore, we believe that executive function and physical impairments might share similar pathophysiology in the progression of disease in PD.

### Limitations

Three limitations of this study must be considered. First, we only recruited patients from one medical center and the findings are, therefore, not representative of the PD general population. Second, we did not record the details of the pharmacotherapy history of these PD patients, especially the possibility of under-treatment with levodopa, which is related to motor functions. Third, the prevalence of frailty might vary according to the different frailty measures used, and the most suitable tool for estimating frailty in PD is still a matter of controversy. Besides, this was a cross-sectional study, which limits the opportunity to analyze the causal associations among disease severity, cognitive changes, and physical frailty in PD patients. Thus, longitudinal studies are warranted for investigating these associations further.

## Conclusion

In summary, we found PD patients with frailty had older age, greater disease severity, and significant deficits in daily activity abilities and most cognitive functions, including attention, executive function, memory, speech, and visuospatial function. Furthermore, severe PD and greater attenuations in executive function could indicate a higher risk of physical frailty in PD. Therefore, our results may help clinicians to screen high-risk PD patients for physical frailty and design efficient intervention strategies to prevent health outcome declines in PD.

## Data Availability Statement

The raw data supporting the conclusions of this manuscript will be made available by the authors, without undue reservation, to any qualified researcher.

## Ethics Statement

The studies involving human participants were reviewed and approved by Institutional review board of Kaohsiung Chang Gung Memorial Hospital. The patients/participants provided their written informed consent to participate in this study.

## Author Contributions

All authors listed have made a substantial, direct and intellectual contribution to the work, and approved it for publication.

## Conflict of Interest

The authors declare that the research was conducted in the absence of any commercial or financial relationships that could be construed as a potential conflict of interest.
